# Smart Device-Driven Corticolimbic Plasticity in Cognitive-Emotional Restructuring of Space-Related Neuropsychiatric Disease and Injury

**DOI:** 10.3390/life12020236

**Published:** 2022-02-04

**Authors:** Kevin B. Clark

**Affiliations:** 1 Felidae Conservation Fund, Mill Valley, CA 94941, USA; kbclarkphd@yahoo.com; 2 Cures Within Reach, Chicago, IL 60602, USA; 3 Domain and Campus Champions Program, NSF Extreme Science and Engineering Discovery Environment (XSEDE), National Center for Supercomputing Applications, University of Illinois at Urbana-Champaign, Urbana, IL 61801, USA; 4 Multi-Omics and Systems Biology Analysis Working Group, NASA GeneLab, NASA Ames Research Center, Mountain View, CA 94035, USA; 5 SETI Institute, Mountain View, CA 94043, USA; 6 NASA NfoLD, NASA Astrobiology Program, NASA Ames Research Center, Mountain View, CA 94035, USA; 7 Universities Space Research Association, Columbia, MD 21046, USA; 8 Expert Network, Penn Center for Innovation, University of Pennsylvania, Philadelphia, PA 19104, USA; 9 Peace Innovation Institute, The Hague 2511, Netherlands and Stanford University, Palo Alto, CA 94305, USA; 10 Shared Interest Group for Natural and Artificial Intelligence (sigNAI), Max Planck Alumni Association, 14057 Berlin, Germany; 11 Nanotechnology and Biometrics Councils, Institute for Electrical and Electronics Engineers (IEEE), New York, NY 10016-5997, USA

**Keywords:** astronaut health, cognitive-emotional restructuring, corticolimbic plasticity, neuropsychiatric disease and injury, smart medical devices, space theragnostic medicine

## Abstract

Escalating government and commercial efforts to plan and deploy viable manned near-to-deep solar system exploration and habitation over the coming decades now drives next-generation space medicine innovations. The application of cutting-edge precision medicine, such as brain stimulation techniques, provides powerful clinical and field/flight situation methods to selectively control vagal tone and neuroendocrine-modulated corticolimbic plasticity, which is affected by prolonged cosmic radiation exposure, social isolation or crowding, and weightlessness in constricted operational non-terran locales. Earth-based clinical research demonstrates that brain stimulation approaches may be combined with novel psychotherapeutic integrated memory structure rationales for the corrective reconsolidation of arousing or emotional experiences, autobiographical memories, semantic schema, and other cognitive structures to enhance neuropsychiatric patient outcomes. Such smart cotherapies or countermeasures, which exploit natural, pharmaceutical, and minimally invasive neuroprosthesis-driven nervous system activity, may optimize the cognitive-emotional restructuring of astronauts suffering from space-related neuropsychiatric disease and injury, including mood, affect, and anxiety symptoms of any potential severity and pathophysiology. An appreciation of improved neuropsychiatric healthcare through the merging of new or rediscovered smart theragnostic medical technologies, capable of rendering personalized neuroplasticity training and managed psychotherapeutic treatment protocols, will reveal deeper insights into the illness states experienced by astronauts. Future work in this area should emphasize the ethical role of telemedicine and/or digital clinicians to advance the (semi)autonomous, technology-assisted medical prophylaxis, diagnosis, treatment, monitoring, and compliance of astronauts for elevated health, safety, and performance in remote extreme space and extraterrestrial environments.

## 1. Introduction

Spaceflight-engaged astronauts must often perform with sustained high physical, mental, emotional, and social proficiency to satisfactorily begin and complete even routine daily mission duties and habitation needs in non-terrestrial environments. Stresses caused by constant extreme environment exposure, such as high-dose cosmic radiation, microgravity, and social isolation or crowding in confined operational locations, exacerbate risks to the health, safety, and wellbeing of astronauts, and further jeopardize already challenging mission goals and outcomes. Expectedly, the 2020 National Aeronautics and Space Administration (NASA) Technology Taxonomy roadmap continues to underscore the need for research and development into, and the application of, technologies and methods that improve human health, life support, and habitation in space and extraterrestrial environments in furtherance of NASA’s goals for manned near-to-deep solar system exploration. Specific areas of interest include, among other categories, (1) medical diagnosis and prognosis, (2) prevention and countermeasures, (3) behavioral health and performance, (4) contactless and wearable human health and performance monitoring, (5) long-duration health, and (6) system transformative health and performance concepts. These areas of emphasis are surveyed here in the context of neuropsychiatric insults, such as mood, affect, anxiety, personality, and psychosis disorders [1,2,3,4,5,6,7,8,9,10], due to the modifications in astronauts’ neurobiology and psychology associated with spaceflight, gateway, and non-terran satellite/planetary surface conditions. In particular, smart theragnostic cognitive-emotional restructuring is considered to be a means of mitigating negative space-induced effects on astronauts’ mental health, wellbeing, and performance. As plans for more short-to-long-duration commercial space events, such as the recent SpaceX civilian Inspiration4 mission, and joint government-industry events, such as those of NASA’s Artemis program, continue to advance, analog and real space investigations need to be conducted to validate the tolerance and effectiveness of these sorts of therapies. Over the next ten years, for example, the Artemis program plans to land the next man and first woman on the Moon’s surface from a lunar orbit Gateway, achieve sustainable human presence on the Moon by 2028, and further provide technologies, capabilities, and business strategies for future successful crewed spaceflight missions to Mars and other destinations. The risks inherent in these activities demand superior attention to astronauts’ mental health and the development and deployment of better methods to surveil, diagnose, and treat health crises in remote space locals.

As with Earth-living scenarios, emotions and the arousing sensations accompanying emotions can weaken or strengthen the storage and retrieval of human memories in space flight and habitation, impacting cognitive, social, and physical performance in relation to the level of intensity and associativity to autobiographical episodes and declarative knowledge. Moreover, extreme emotional experiences and resultant memories may become maladaptive, causing neuropsychiatric problems whether they vividly enter or remain inaccessible to human consciousness, leaving astronauts vulnerable to suboptimal mental health outcomes in non-terran environs. Lane et al. [11] and other experts (e.g., [12,13]) have synthesized appealing, albeit somewhat preliminary, psychotherapeutic programs now being further investigated by (Earth) clinicians that rely on selectively reconsolidating patients’ integrated memory structures. The modification of these structures, comprising (implicit/explicit) emotional responses, autobiographical memories, semantic schema, and other cognitive structures, will help clinicians better understand and treat mood, affect, and anxiety disorders, as well as consequences from traumatic brain injury. Some of these programs, which may render good prevention, intervention, and postvention techniques for astronaut mental health, aim to apply the Yerkes-Dodson law of arousal-modulated performance within (mental/physical) situational contexts to (1) recover or uncover patient memories of emotional traumas, and (2) to associate and then reconsolidate corrective cognitive representations and emotional responses with the same target memories through appropriate use of a single psychotherapeutic modality or various modality combinations (i.e., behavioral, cognitive-behavioral, emotion-focused and/or psychodynamic/psychoanalysis approaches).

While this type of technique is inspired by well-established clinical and basic neuroscience research findings on memory modulation [14,15,16,17,18,19,20,21,22,23,24], the concept in its present form is beset by methodological imprecision for Earth-based clinical efficacy and compliance. Such problems are common in other purely psychotherapeutic strategies, and integrated memory structure rationales might only find, even after revision, the greatest clinical success in patients presenting medically tractable mild to moderate neuropsychiatric symptoms. Problems for clinical success are further exacerbated by situational difficulties in finding and administering neuropsychiatric diagnosis and treatment solutions to astronauts. To overcome such gaps in medical prophylaxis, diagnosis, treatment, monitoring, and compliance, psychotherapeutic approaches may be suitably optimized with the co-application of repurposed, newly emerging, and next-generation smart computer-interfaced theragnostic medical devices linked to assistive telemedicine technologies and medical social robot or virtual digibot therapists. Trends in state-of-the-art theragnostics (Box 1) will likely benefit astronaut mental health prognosis in space and extraterrestrial environments, as well as in terran environments following the return of astronauts to Earth. Advantages from these improved theragnostic strategies also may be applied to help innovate changes in healthcare on Earth for civilian populations exposed to increasing challenges (e.g., extremer Earth environments, longer human lifespans, denser human populations and overcrowding, heightened human de-socialization or conflict, etc.), which may worsen mental health in a demographic group-dependent and/or -independent manner. Past translational medicine successes, such as NASA’s involvement in bringing telemedicine approaches to Earth-based medicine and underserved communities, may serve as models to transform current trends in these areas, resulting in more economical, accessible, and effective Earth medical policies and practices.

Box 1 **Highlights.**

- Memories of extreme emotional experiences may produce psychiatric problems whether they vividly enter or remain inaccessible to consciousness in Earth and non-terran environments, causing health, wellbeing, and performance decrements in dangerous contexts.
- Newer psychotherapeutic programs for the selective reconsolidation of traumatic patient memories aim to modulate vagal tone to treat mood, affect, and anxiety disorders, and may be useful in treating space-induced neuropsychiatric illness and injury.
- Though neuroscience research findings support these types of intervention, the program in its present form is beset by methodological imprecision.
- Optimizing therapeutic efficacy by combining these programs with the personalized advantages offered from the use of minimally invasive smart neuroprosthetic technologies, such as brain stimulation methods, improves clinical options, but needs further study in humans and animal models.
- Such technologies drive vagal activity, corticolimbic plasticity, and the cognitive–emotional restructuring of patients suffering from psychiatric symptoms of varying severities and pathophysiologies.
- Current and future state-of-art must adopt seamless integrated and interoperable systems with open- and closed-loop capabilities that enable remote human and (semi)autonomous robotic and virtual digibot clinicians for better monitoring and treatment of inflight astronaut patients.
- Medical governing bodies must establish best policies and practice guidelines for the ethical design and use of these technological systems on Earth and beyond.

## 2. The Problem of Optimal Cognitive-Emotional Restructuring

Typical countermeasures for poor astronaut mental health involve physical exercise, cognitive recreation, and socio-emotional supports. However, neurotechnology and cotherapy alternatives developed for Earth inhabitants may extend to space travelers, although more research needs to be conducted to determine how non-terran environments may alter the safety and efficacy of such treatment strategies. Alternative neuropsychiatric approaches noted above neglect how ground and flight clinicians or emerging smart medical social robots or digibots (cf. [25,26]) can achieve closer to optimal therapeutic efficacy. Current examples of affordable, accessible digital devices with high therapeutic potential include the Paro, eBear, Kaspar, Nao, and RoboTherapy companion bots and Tess, Sara, Wysa, and Woebot digibots. These interactive meta-learning inferential devices, which interface with patients via translatable speech and text communications, represent sound remote (semi)autonomous smart assistive surrogate healthcare solutions for patients expressing dysfunctional mood and affect symptoms caused by social isolation and stress, similar to those associated with space mission work and living conditions. However, they remain untested for many psychotherapy approaches, and their degree of effectiveness can be improved. One major strategy is to incorporate digitally delivered psychotherapy and integrated memory structure rationales with the advantages offered by minimally invasive smart neuroprosthetic technologies, such as (1) vagus nerve stimulation (VNS), (2) transcranial electrical stimulation (TES), (3) transcranial near-infrared stimulation (TIRS), (4) transcranial magnetic stimulation (TMS), and (5) emerging smart pharmaceuticals, such as microRNA-targeting mimics, antagomirs, and micro-/nanosized drug delivery, regenerative, and surgical platforms [27,28,29,30,31,32,33,34,35,36,37,38,39]. Many of these smart medical technologies are either already commercially available or undergoing reduction to practice for the control of mood, affect, and anxiety disorders that are currently resistant to conventional drug and/or psychotherapeutic techniques. In some of the most severe psychiatric conditions, the oftentimes labile, progressive, and irreversible expression of forebrain pathophysiologies renders patients unresponsive to otherwise known safe (and tolerable) disease prophylaxes, management, and treatments. Even Earth patient populations suffering from mild to moderate psychiatric disorders, such as tiered post-traumatic stress disorders and chronic depression, respond to psychotherapy, chemotherapy, or joint psychotherapy/chemotherapy with significant chronic and acute symptom relapse rates [40,41]. Integrated memory structure rationales seek to improve upon the effectiveness of standard psychotherapy for patients showing complex emotional components, especially as it limits the problematic heterogeneity of psychotherapy choice and administration (e.g., poor psychotherapeutic entry points, client-clinician rapport, patient compliance, trauma identification and schema restructuring, etc.). Yet, many clinicians in support of integrated memory structure rationales notably fail to detail methods for determining and eliciting suitable, consistent therapeutic (i.e., moderate) levels of patient arousal in therapeutic environments. They also often fail to fully account for diagnoses for which these programs may yield less than marginal efficacy, such as cognitive-behavioral impairments accompanied by serious persistent corticolimbic structural and functional abnormalities.

## 3. Smart Solutions for the Problem of Optimal Cognitive-Emotional Restructuring

The process of reactivating and reforming memories to accomplish cognitive-emotional restructuring in patients demands, in theory and practice, very controlled physiological states of healthy or (remedied) afflicted nervous systems. Cutting-edge smart precision medicine may provide space medicine practitioners with much needed clinical tools to resolve such flaws in implementing integrated memory structure rationales and, therefore, to increase their therapeutic potential. Smart neuroprosthetics and drug delivery systems, acting via highly selective substrate and temporal targeting of disease-affiliated molecules, cellular organelles and pathways, brain networks, and nerve fiber groups, can be employed to overcome many neuropathologies observed in the hippocampus, amygdala, and medial prefrontal and cingulate cortices (cf. [14,28,32,33,42,43,44,45]). Such pathologies include, among other changes, (1) disturbances in neuro- and synaptogenesis, (2) protein expression, (3) neuronal excitotoxicity and glial proliferation, and (4) glucocorticoid-dependent neurotrophic factor concentrations, which are connected to mood, affect, and anxiety disorders and hypoxic and ischemic brain damage. Besides compensating comorbid neurological states, these advanced technologies also effectively alter patient learning, memory, and executive function (e.g., [14,15,31,46,47,48]) through targeted neuroplasticity training. Targeted neuroplasticity training modulates brain synaptic plasticity (e.g., [14,44,45,49,50]), single-unit and field potential phasic activity (e.g., [14,51,52]), the signal-to-noise ratio of neurotransmission (e.g., [14,53]), and neurotransmitter synthesis, release, and re-uptake (e.g., [14,54,55]) to effect changes in human and animal disease outcomes and performance.

## 4. Cognitive-Emotional Restructuring via Vagus Nerve Stimulation

**Figure 1:**
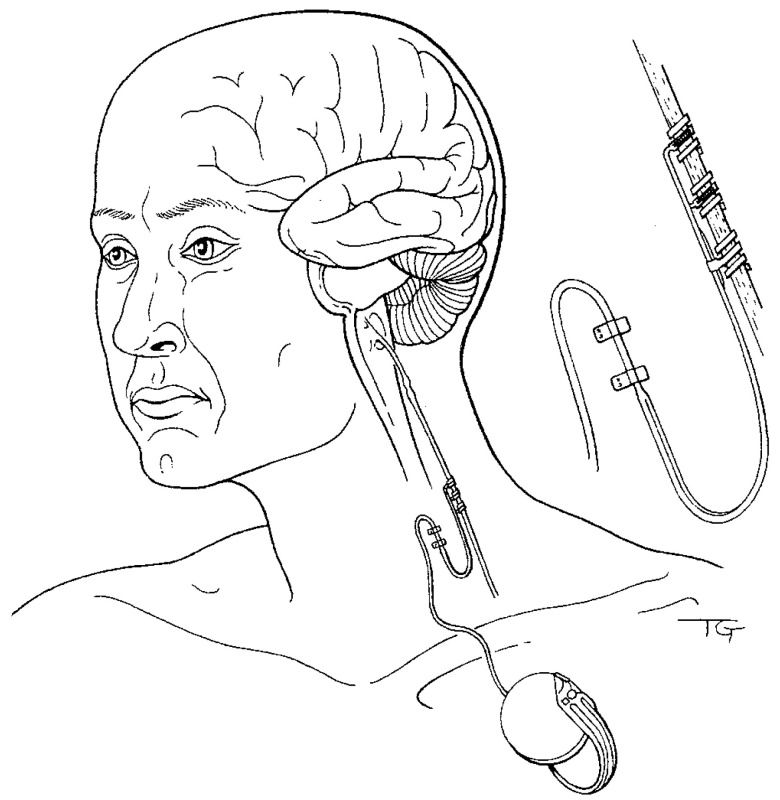
Illustration of implanted vagus nerve neurocybernetic prosthesis used to treat patients suffering from drug-unresponsive epilepsy, depression, and affect disorders. Placement of helical electrodes on the left cervical vagus nerve, with intermittent stimulation provided by a small remotely controlled programmable device implanted subcutaneously in the upper chest, manages side effects while delivering safe, efficacious therapy. Most patients are stimulated at maximally tolerated intensities of around 1 mA and frequencies of 20–30 Hz, with a stimulation cycle of 30 s on, and 5 min off. These stimulation parameters enhance memories in neurologically compromised human patients. Less invasive transcutaneous electrical and magnetic vagus nerve stimulation and administration technologies (not shown) also effectively treat psychiatric disorders with beneficial cognitive effects, and provide commercially available portable solutions ready to be deployed in non-terran environments to treat astronauts with mental health conditions. Figure 1 reproduced from [14] with permission.

In the case of VNS (Figure 1), which mimics the viscerosensory information associated with emotional events and arousal critical to integrated memory structure rationales, corticolimbic plasticity is generated from titrated, feedback-controlled stimulation parameters that fall well below the threshold for the maximal activation of unmyelinated vagal C fibers. Although the vagus nerve is comprised of myelinated and unmyelinated afferents, subthreshold stimulation parameters produce moderately arousing (implicit/explicit) cardiopulmonary neural signals that are carried by myelinated afferents to the brain. Such signals exert widespread polysynaptic control over brain function and behavior (cf. [55]), including replicating the Yerkes-Dodson inverted-U pattern of memory-modulated performance in humans and animals [14,15,46,47]. Memory performance changes correlate with respective protracted increments and decrements in the hippocampus and medial prefrontal cortex pyramidal cell responses characteristic of bioamine-dependent long-term potentiation and depression [14,49,50]. These brain areas and processes are known to be impacted by space travel. Further VNS-induced plasticity manifests in beta- [46,51], theta- (cf. [14,56]), and gamma-band [14,52] spectral power alterations in the corticolimbic field potentials thought to be essential for encoding, consolidating, retrieving, and reconsolidating autobiographical emotional information and semantic schema. While these VNS findings are largely taken from protocols typically delivered through surgically implanted programmable devices, similar results are predicted for protocols delivered through more noninvasive, transcutaneous electrical and magnetic nerve stimulation (cf. [57,58]). Optimal technological feasibility and the effectiveness of these and other newer minimally invasive techniques, in Earth and in space and extraterrestrial environments, depend on ongoing advances in wearable wireless nervous system-computer interfaces to automatically detect and initiate real-time responsive neurostimulation for psychiatric crises (cf. [59]). Additionally, as with other precision medical technologies, their exact reproducible application surpasses the psychotherapeutic value of older alternative techniques, such as generating arousal from hand-held dynamometers, and may be paired, for instance, with emotion-laden content of (computer-presented) stimuli or additional methods to adjust disorder-compromised vagal tone. Astronauts experience vagal tone perturbation under physical and emotional stress and, therefore, are superb candidates for VNS-aided cognitive-emotional restructuring to remedy any possible mood, affect, and anxiety disease severity caused by microgravity and work conditions.

### 4.1. Anatomy and Afferent Pathways of the Vagal System

An examination of the anatomy and afferent pathways of the vagal system serves to identify the biomechanisms contributing to VNS-induced cognitive-emotional restructuring (cf. [14,55]) on Earth or beyond [1]. Located in the brainstem, the nucleus of the solitary tract and the parabrachial nucleus receive most of the vagal afferent fibers projecting from the nodose and jugular ganglia [60,61,62]. The topographically organized nucleus of the solitary tract relays the majority of vagal afferents, sending diffuse projections to all rostral levels of the brain. The nucleus of the solitary tract’s targets include the midbrain nuclei critical for the neuromodulation of brain processes and forebrain areas importantly involved in cognition, emotion, and sensory integration (cf. [63,64]). For example, the locus coeruleus, the major collection of norepinephrine cell bodies in the brain, receives indirect nucleus of the solitary tract input via the paragigantocellularis and the perifascicular area of the prepositus hypoglossi (cf. [65]). Additional major bioamine systems receiving direct nucleus of the solitary tract innervation include the dopaminergic ventral tegmental area and medial substantia nigra [66], and the serotonergic dorsal raphé nucleus and periaqueductal gray area [67,68]. Through these and other neuromodulatory systems, the vagal system indirectly and widely impinges on forebrain areas. However, ascending fibers of the nucleus of the solitary tract also directly innervate the hippocampus, the central and medial nuclei of the amygdala, and other areas [62,66,69]. Additional indirect nuclei of the solitary tract projections, from the parabrachial nucleus or thalamus, impinge on the hippocampus, amygdala, medial prefrontal cortex, and insular cortex, the primary viscerosensory cortex (cf. [64,70,71]).

### 4.2. Neurochemistry of the Vagal System and Its Ascending Targets

As noted above, the vagal system, via the nucleus of the solitary tract and the parabrachial nucleus, has the capacity to modify cognition and emotion through indirect and direct influences over forebrain physiology. Vagal afferent activity exercises particularly strong indirect control of forebrain function via the bioamine systems central to the expression of psychiatric illnesses. Work by the author [14,53] and other researchers (cf. [72,73,74,75]) demonstrate that vagal activation inhibits, then potently excites coerulear neuronal firing rates in laboratory rodents, presumably increasing microdialysis-measured norepinephrine release in the hippocampus, amygdala, and neocortex [45,54]. Seemingly contrary evidence, however, has failed to confirm elevated levels of the catecholamine precursor 3,4-dihydroxyphenylalamine or DOPA in rat forebrains following VNS administration [14]. Therapeutically effective VNS instead elevated 3,4-dihydroxy-phenylalamine concentrations in the locus coeruleus [14], findings that indicate that vagal stimulation likely initiates the manufacture of catecholamine-containing storage vesicles and, therefore, norepinephrine synthesis in the cell bodies of coerulear projection neurons. Consistent with this interpretation, 3,4-dihydroxyphenylalamine turnover was probably too small to be detected in the presynaptic terminals located in the hippocampus, amygdala, and neocortex. Because 3,4-dihydroxyphenylalamine accumulation represents increases in both norepinephrine and dopamine synthesis, small increases in 3,4-dihydroxyphenylalamine concentrations in additional brain regions which receive substantial dopaminergic innervation, such as the caudate nucleus, might likewise have gone undetected. Nevertheless, catecholamine synthesis, undetected by turnover measurements, may still sufficiently restore ‘normal’ corticolimbic function in psychiatric patients by augmenting neuronal activity and by reversing damaging cytological effects due to stress and steroidal hormones (cf. [76,77]) and other causes (e.g., [78]). In contrast to catecholamines, the findings imply that vagally activated raphé projection neurons help alleviate symptoms of depression. For example, raphé nuclei inactivation reduces several different VNS effects, such as antinociception [79]. Additionally, the widespread depletion of brain serotonin impairs VNS-induced seizure suppression [80]. Dramatically raised concentrations of 5-hydroxy-indoleacetic acid or 5-HIAA, a marker for serotonin activity, occur in epileptic patients treated with VNS [81]. Furthermore, the delivery of VNS to rats slightly increases forebrain turnover of the immediate serotonin precursor, 5-hydrotryptophan or 5-HTP, indicating elevated serotonin synthesis. Changes in these respective serotonin metabolites and precursors possibly account for trends in altered medial frontal cortex synaptic plasticity and neuronal signal-to-noise ratios consistent with serotonin release [14]. VNS, therefore, demonstrates some capacity to affect serotonin activity and the function of raphé nuclei targets, possibly restoring balance to corticolimbic and corticocortical circuits in epileptic and depressed patients (cf. [57,58,82]). Such activation likely increases the synthesis and release of serotonin in brain areas, including the hippocampus and medial frontal cortex, which are important for the expression of learning, memory, and psychiatric disorders on Earth and non-terran environments. Comparative studies involving animal models and humans exposed to extreme space conditions, such as microgravity and high-dose radiation, must verify these findings for the suitable prophylaxis and treatment of space-related neuropsychiatric injury and disease [1].

### 4.3. Plasticity of Hippocampus and Medial Frontal Cortex Evoked Population Responses

**Figure 2:**
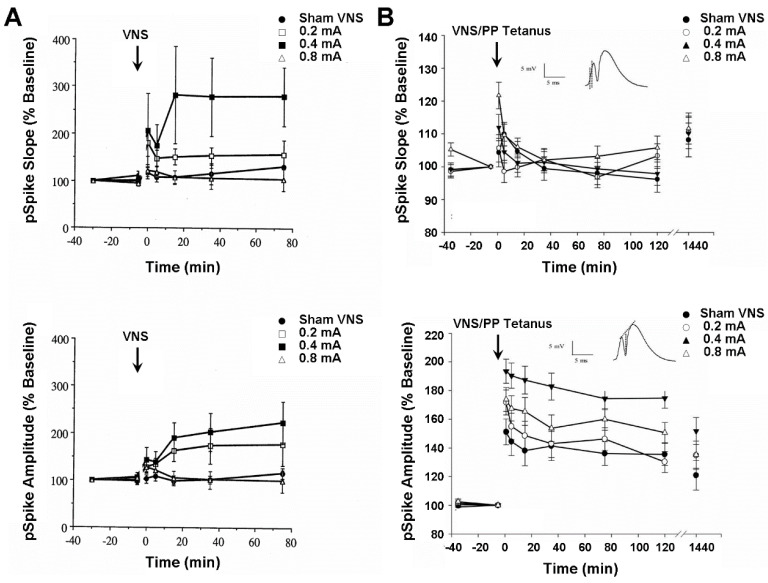
VNS-induced neuronal plasticity of the hippocampus, a brain area affected by spaceflight and important for cognitive-emotional restructuring. Activity from neuron population responses in anesthetized (panel (**A**), top and bottom displays) and awake, freely moving laboratory rodents (panel (**B**), top and bottom displays) were recorded from the dentate gyrus of the hippocampus before and after VNS at 0.2, 04, or 0.8 mA and 20 Hz for 30 s. Figure 2A and Figure 2B are reproduced, respectively, from [14,50] with permission.

Besides controlling mood and seizure expression, VNS also modulates learning and memory performance in both humans [15] and laboratory rats [46,47]. The author tested whether these VNS effects might be reflected in the neuronal activity of the hippocampus and medial frontal cortex. These two structures are notably involved in the expression of learning, memory, epilepsy, clinical depression, and hypoxic and ischemic brain injury on Earth [14], as well as space-related brain alterations affecting cognitive function and mental health [1,2,3,4,5,6,7,8,9,10]. In the first set of experiments, perforant path-evoked multiunit potentials from the ipsilateral dentate gyrus of the hippocampus were recorded from anesthetized rats before and after shamVNS or left-cervical VNS was delivered at one of three intensities (i.e., 0.2, 0.4, and 0.8 mA). The VNS delivered at a moderate intensity produced an initial probable post-tetanic potentiation, followed by a slight depression, perhaps a paired-pulse inhibition, that rapidly reversed into a significant and protracted increase (280%) in the mean percent change from the baseline of the dentate gyrus population spike (pSpike) slope and amplitude (Figure 2A). A similar, but more transient, mean increase (160%) in the pSpike slope and amplitude also occurred following the VNS given at the low intensity, whereas the group of animals receiving high-intensity VNS only exhibited a nonsignificant trend of relatively minor (120%) short-term increases in pSpike slope and amplitude. It remains unclear why higher intensity stimulation failed to produce a more pronounced effect. No significant changes occurred in the population excitatory postsynaptic potential (EPSP) slope for any stimulus intensity. The highly labile long-term elevation of the pSpike slope and amplitude, a somal/axonal response, without concurrent changes in population EPSP slope, a dendritic response, characterizes norepinephrine-dependent long-term potentiation of dentate gyrus cells [14]. These results, and comparable ones from awake animals (Figure 2B), therefore suggest that VNS might induce plastic and metaplastic changes in hippocampal neuronal activity by eliciting the release of norepinephrine from the locus coeruleus. Such changes possibly compensate for a decreased hippocampal volume associated with clinical depression by improving hippocampal output to target structures, such as the medial frontal cortex and anterior cingulate gyrus. This combination of effects likely enhances the capabilities of psychotherapy and VNS co-treatment, such as integrated memory structure rationales, of Earth and space-induced neuropsychiatric disorders, and need to be studied further for remote mental healthcare deployment in space travel and habitation contexts.

**Figure 3:**
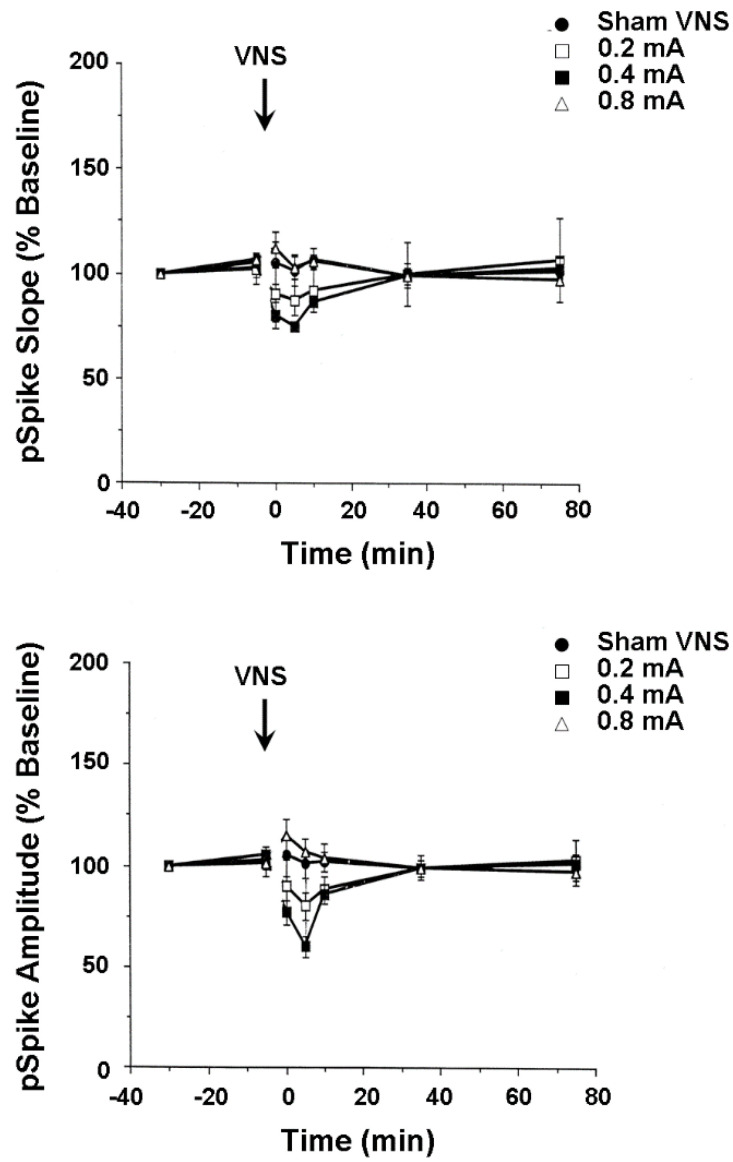
VNS-induced neuronal plasticity of the medial frontal cortex, a brain area affected by spaceflight and important for cognitive-emotional restructuring. Activity from neuron population responses in anesthetized rats (top and bottom displays) were recorded from the infralimbic area of the medial frontal cortex before and after VNS at 0.2, 04, or 0.8 mA and 20 Hz for 30 s. Figure 3 reproduced from [14] with permission.

In the second set of experiments, mediodorsal thalamus-evoked population responses were recorded from the medial wall of the infralimbic area of the medial frontal cortex in anesthetized rats before and after shamVNS or cervical-level VNS delivered at one of three intensities (i.e., 0.2, 0.4, and 0.8 mA). A nonsignificant trend toward a moderately lasting mean percent decrease (35%) from baseline in the medial frontal cortex pSpike slope and amplitude followed VNS delivered at both low and moderate intensities (Figure 3). Reported evidence from other laboratories indirectly suggests that this effect might result from the VNS-induced release of dopamine from the ventral tegmental area and serotonin from the dorsal raphé nucleus. Both dopamine and serotonin depress medial frontal cortex synaptic activity [51], while serotonin inhibits medial frontal pyramidal cell firing rates [50]. However, dopamine [54,55] and serotonin [52,53] also paradoxically facilitate the incidence of medial frontal cortex long-term potentiation, depending on the areal and laminar myeloarchitecture, cytoarchitecture, and receptor pharmacology. Though speculative, the above small effect possibly indicates that VNS activates the ventral tegmental area and dorsal raphé nucleus neurons which, in turn, respectively release dopamine and serotonin onto medial frontal cortex pyramidal cells, impairing their capacity to transmit information. Such changes perhaps compensate for the increased reactivity of certain medial frontal cortex areas during the early stages of clinical depression, restoring functional connectivity with other limbic circuit structures, such as the anterior cingulate gyrus and hippocampus. As with findings from the hippocampus, these results indicate biomechanisms that underlie the possible successful treatment of astronaut neuropsychiatric conditions with integrated memory structure rationales and VNS.

### 4.4. Activity of Hippocampus and Medial Frontal Cortex Single Neurons and Local Field Potentials

**Figure 4:**
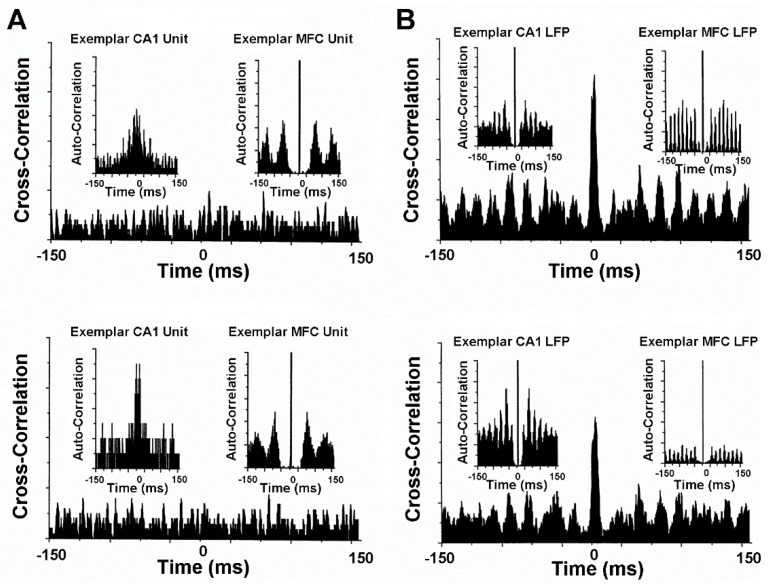
VNS-induced correlated activity of corticolimbic structures, areas affected by spaceflight and important for cognitive-emotional restructuring. Activity from single-unit (panel (**A**), top and bottom displays) and local field potential (panel (**B**), top and bottom displays) pyramidal neurons in the CA1 area of the hippocampus and from the infralimbic area of the medial frontal cortex (MFC) were recorded from anesthetized laboratory rodents before and after VNS at 04 mA, 20 Hz for 30 s. In each panel, the top and bottom displays show pre- and post-VNS, respectively. Figure 4 reproduced from [14] with permission.

**Figure 5:**
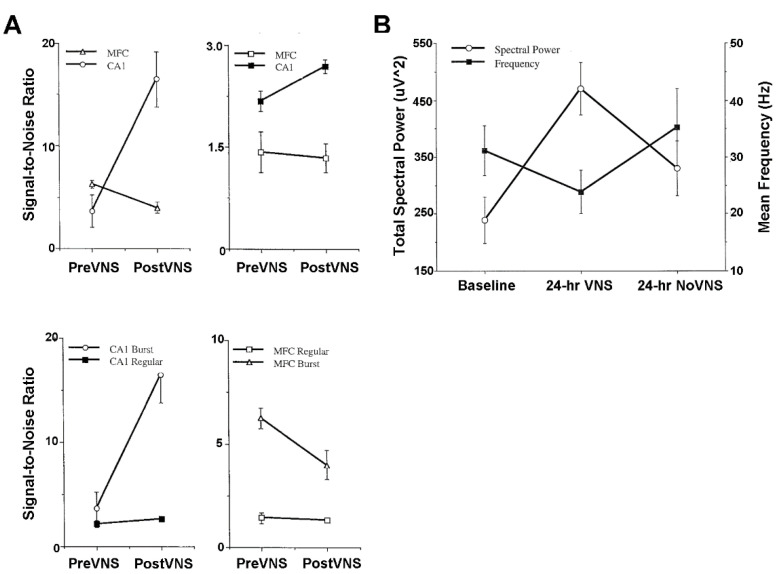
VNS-induced increases in the power of neuronal activity in corticolimbic structures affected by spaceflight and important for cognitive-emotional restructuring. VNS, delivered at 04 mA, 20 Hz for 30 s to anesthetized laboratory rodents, increases the signal-to-noise ratio of CA1 pyramidal (Burst) and inhibitory (Regular) neurons recoded from the hippocampus, while simultaneously decreasing the signal-to-noise ratio of infralimbic area pyramidal (Burst) and inhibitory (Regular) neurons recorded from the medial frontal cortex (MFC) (panel (**A**), left top and bottom displays compare respective pyramidal and inhibitory neuron output). Consistent with these findings, VNS administered for 24 h at therapy-relevant continuous intermittent parameters markedly increases the total spectral power and decreases the frequency of frontal cortex electroencephalogram activity recorded from awake, freely moving rats (panel (**B**). Figure 5A and Figure 5B, respectively, reproduced from [14,51] with permission.

Animal model studies evaluating simultaneously recorded single-unit, multi-unit, and local field potential (LFP) activity from the CA1 area of the hippocampus and from the infralimbic area of the medial frontal cortex before and after VNS provide further neurophysiological insights into treatment mechanisms [14]. These mechanisms will need to be carefully scrutinized to determine the validity and power of prospective neuropsychiatric treatment courses in space and extraterrestrial environments [1,2,5,6,7]. The CA1 and medial frontal cortex are particularly good forebrain areas in which to study the oscillatory and synchronous activity associated with learning, memory, and neuropsychiatric disorders. Gamma frequencies in both areas are thought to be importantly involved in the encoding of sensory information and, therefore, are critical to integrated memory structure rationales and VNS co-treatment. Comparisons between pre-VNS and post-VNS auto-correlograms of bursting pyramidal cells simultaneously recorded from CA1 and the medial frontal cortex show diametrically different effects on neuronal discharge (Figure 4A). VNS increased the number and tightened the distribution of the auto-correlated events (i.e., spikes) of synchronous, non-oscillatory CA1 cells, whereas VNS decreased the number and broadened the distribution of auto-correlated events of non-synchronous, oscillatory medial frontal cortex cells. No discernable changes occurred for the cross-correlational activity between these cell distributions. Similar diametrically different VNS-induced modifications were observed from the auto-correlations of gamma LFP activity recorded from CA1 and the medial frontal cortex during unit activity (Figure 4B). VNS increased the number of auto-correlated events and shifted the oscillatory register to slightly higher gamma frequencies in CA1. Conversely, VNS decreased the number of auto-correlated events and shifted the oscillatory register to slightly lower gamma frequencies in the medial frontal cortex. Moreover, cross-correlational analysis between CA1 and medial frontal cortex LFP activity showed a slight decrease in circuit synchrony between the two sites, as evidenced by a smaller central peak. Collectively, these findings suggest that VNS increases hippocampal output and simultaneously decreases medial frontal cortex output, perhaps restoring limbic circuit balance to the pathologically altered brains of depressed patients and patients experiencing other neuropsychiatric conditions. This notion is further supported by the VNS-induced augmentation of the signal-to-noise ratios of CA1 and medial frontal cortex pyramidal cells [14] (Figure 5A). VNS produces a bioamine-like increased amplitude modulation of CA1 pyramidal cell spikes (cf. [27]), and a more modest decreased amplitude modulation of medial frontal cortex pyramidal cell spikes (cf. [51]). The increased efficiency of classical information transmission by CA1 pyramidal cells, as measured with information-theoretic parameters [14], possibly compensates for the decreased hippocampal volume associated with depression. Additionally, the decreased efficiency of classical information transmission by medial frontal cortex pyramidal cells possibly compensates for the regionally specific medial frontal cortex hypermetabolism associated with depression. Such changes in neuronal excitability are further supported by observed frontal cortex convexity multicellular plasticity during VNS [51], which shows significant total power spectral density increases and shifts from fast to slow beta frequencies, likely due to the recruitment of silent neurons (Figure 5B).

## 5. Alternative Cognitive-Emotional Restructuring via Transcranial Magnetic Stimulation

Use of VNS to enhance cognitive-emotional restructuring in astronauts is attractive for its more-or-less straightforward biological relationship to the formation and retrieval of traumatic emotional memories. TMS, another potentially efficacious treatment adjuvant, may also facilitate patient cognitive-emotional restructuring by directly improving or impairing neural communication and the (structural and functional) connectivity between the limbic and cortical areas, contributing to memory consolidation and retrieval (cf. [83,84,85,86,87,88]). In comparison to VNS, the corticolimbic plasticity induced by precise TMS alone should be considered to be less representative of the naturally occurring bioprocesses and brain changes accompanying emotional experiences, since TMS bypasses the recruitment of the vagal system. Nevertheless, TMS has been shown to be a credible means for modifying the storage capacity and accuracy, retrieval, and/or reconsolidation of emotional memories in animal models of anxiety, and in humans suffering from real-life emotional traumas or enduring experimentally constructed emotional situations (e.g., [89,90,91,92,93,94]). The impressive successes of TMS for altering memory attributes and performance levels specific to (1) patient sensorial/perceptual modalities, (2) patient gender, (3) degree of patient attention, and (4) emotional, semantic, and procedural content of patient memories [95,96,97,98,99,100,101] has encouraged its application for the treatment of (1) major depression, (2) dementia, (3) post-traumatic stress disorder, (4) schizophrenia, and (5) other psychiatric conditions (e.g., [102,103,104,105,106,107,108,109]). Though the targeted phenomenological effects of TMS on patient memory and cognition, and therefore psychiatric status, largely result from physiological tuning and the reorganization of lower-band brain function, often through large-scale network interference or facilitation (cf. [109,110,111,112,113,114]), the cytological and biochemical mechanisms mediating such plasticity are only now being elucidated. Preliminary sets of findings indicate that TMS effects changes in synaptic transmission, as well as corresponding synaptic structure and density, in an intensity-specific manner, similar to VNS, and that such changes result from altered brain concentrations of (1) amino acid and bioamine neurotransmitters, (2) neurotrophic factors, and (3) protein kinase-dependent protein synthesis, transcription, and cell metabolism and growth [115,116,117,118,119,120]. Taken together, these and additional findings indicate that the combined use of TMS with psychotherapeutic strategies provides a safe, efficacious way to effect cognitive-emotional restructuring in Earth patients (e.g., [121]) and perhaps astronauts.

## 6. Nervous System-Computer Interfaces, Medical Robots and Digibots, and the Future of Seamless Integrated Device-Driven Theragnostic Cognitive-Emotional Restructuring

Psychotherapeutic integrated memory structure rationales for the corrective reconsolidation of traumatic arousing or emotional experiences, autobiographical memories, and semantic schema are predictably enhanced by the co-application of cutting-edge smart precision medicine. Combining these rationales with neuroprosthesis-driven VNS or TMS, for example, allows clinicians to selectively modify vagal tone and brain processes associated with psychiatric trauma for more personalized, optimal, noninvasive treatment of mood, affect, and anxiety disorders. Choices among commercially available small portable stimulation and wearable monitoring devices designed for Earth-based clinical treatment of various neuropsychiatric and motor indications permits existing neurotechnologies to be expediently and cheaply adapted for space medicine purposes, without incurring high payload and health, life support, and habitation systems burdens. Although legal, regulatory, and ethical issues remain for Earth medical policies and practices (e.g., [25,26,122,123]), these sorts of devices may be equipped with minimally invasive contact or contactless sensor/stimulator-connected computer interfaces, which can be programmed with user-friendly proprietary software. In addition, these systems may be linked with (ultraparanoid computing) encrypted semi-autonomous or autonomous mobile or stationary virtual remote medicine systems to diagnose, treat, and monitor patients based on a range of smart real-time biomarker data analytics and electronic record keeping (e.g., [59,124,125,126,127]), including, but not limited to, those associated with typical physiological vital signs, brain function, and continuous body fluid chemical profiles.

Despite these space deployment advantages, psychotherapeutic methods require clinician-completed patient assessments, such as industry-standardized semi-structured and structured inventories, and clinician-guided therapy sessions to maximize patient outcomes. In the absence of human clinical practitioners, neurotechnologies must seamlessly and interoperably integrate smart human-emulating social medical robot or virtual digibot therapists to execute interactive psychotherapy in coordination with the operation of neurocybernetic prosthetic systems. Considerable amounts of work over many decades continue to refine and improve artificially intelligent, deep and meta-learning inferential digital therapists, resulting in varying positive mental health and wellbeing impacts on patient populations from pediatric to geriatric cohorts (e.g., [25,26,122,123]). These technologies, which may be telemedically linked via neurocybernetic prosthetic command and control systems for optimal VNS or TMS delivery, are represented by successful findings noted for the previously discussed social robots and digibots [122]. That said, progress remains slow in this area, and research and development need to concentrate on increasing the safety, efficacy, and clinician-patient rapport of digital therapists for the best patient outcomes on Earth and in environments beyond Earth. Some current identifiable gaps in the state-of-the-art systems involve determining and using more powerful, internally and externally valid, and precise psychological, biological, behavioral, and digital biomarkers as well as more reliable and accurate fast theragnostic computational models that better manage data workloads/-flows for superior patient therapy experiences and personalized mental health diagnosis and monitoring. Promising disease marker examples include constellations incorporating acoustic, structural, and semantic language production, use, and comprehension to detect the onset and severity of neuropsychiatric conditions [128,129,130,131,132,133,134], whereas promising computational model examples include feature classifier/extraction/prediction algorithms capable of adaptive quasi-model-free/-based neural net embedding, trainable distributed cognition-emotion mapping, and artificial (surrogate therapist) personality trait parameterization for machine intuitive causal physics and psychology, compositionality and learning-to-learn, and efficient real-time gradient descent deep learning and thinking [25,133,134]. Many of these artificial intelligence and machine learning milestones continue to be emphasized in competitive Earth-based biomedicine/-technology commercial sectors, and may be realistically achieved over the next five to eight years with safe, efficacious deployment for suitable space and extraterrestrial utility within the decade [134].

## 7. Concluding Remarks about Medical Ethics

Dedicated industry, academia, and government research and development will greatly advance these emerging technologies and their clinical translation over the next ten years, including the successful integration of artificial surrogate-mediated theragnostic approaches with smart interoperable pharmaceutical and other treatment delivery systems [1,133]. Suitable government and industry regulation of technology safety, efficacy, privacy, and security must keep pace with that future. Rigorous transparent discussion about proper technology-assisted medical information management and use are essential to the smooth, high-quality deployment of intelligent artificial surrogate clinicians for both Earth and space medicine purposes. Such large-scale coordinated community efforts facilitate cultural understanding about the medical and economic value and the societal role of intelligent technologies, encouraging and sustaining consumer confidence and positive relations between patients and healthcare resources and services providers. To that end, clearer, enforceable guidelines [25,26,121,122,123,132,133,134,135] need to be constructed to determine whether and what artificially intelligent surrogate clinician technologies should be subject to standard healthcare technology regulatory evaluation and approval. Guidelines may address (1) technology use outside the supervision of healthcare professionals, (2) professional organization recommendations and the establishment of best policies and practices for training healthcare providers for technology use in different healthcare models, (3) satisfying duties of care, reporting of harm, and issuing reliable pathways for risk assessment and services referral, (4) technology oversight and services transparency which respect patient autonomy, vulnerability, and privacy, and (5) the scrutiny and mitigation of institutionalized and individualized biases that lead to unwanted technology and services delivery inequities. Inequities may include (1) the status of theragnostic validation, (2) the availability of open-source platforms for obtaining and distributing digital biomarker data, (3) the appropriateness of behavioral and digital phenotyping, (4) the comprehensiveness of data-driven learning engines, (5) the use of real-world evidence, (6) the cost and infrastructure for data storage and analysis, (g) seamless data integration with clinical records, and (7) policed data ownership and release.

## 8. Contributions to the Field Statement

Unprecedented competition and cooperation between public and private sector segments of the space industry now motivate new technological trends to support the emerging era of deep space human exploration and habitation, as exemplified by the lunar Artemis program and planned future Mars expeditions. The application of cutting-edge smart precision medicine is one significant area for crewed mission development and deployment, with the aim of creating best remote theragnostic practices and technologies to help reduce the incidence and severity of astronaut physical and mental health risks incurred from extreme space and extraterrestrial environment exposure. Newer psychotherapeutic and neurotechnology cotherapies provide powerful clinical and field/flight situation methods to selectively modify space-altering vagal tone and corticolimbic plasticity, optimizing the cognitive-emotional restructuring of astronauts suffering from space-related neuropsychiatric disease and injury, including mood, affect, and anxiety symptoms. Such treatment protocols, when combined with human clinician-operated telemedicine and/or (semi)autonomous (digi)robotic medical solutions, will reveal deeper insights about the mental illness states experienced by astronauts and about necessary research, development, and ethical space medicine directions for improved astronaut health, safety, and performance during solar system travel and habitation over the next decade and beyond.
